# Proposal and Thermodynamic Assessment of S-CO_2_ Brayton Cycle Layout for Improved Heat Recovery

**DOI:** 10.3390/e22030305

**Published:** 2020-03-06

**Authors:** Muhammad Ehtisham Siddiqui, Khalid H. Almitani

**Affiliations:** Mechanical Engineering Department, King Abdulaziz University, Jeddah 21589, Saudi Arabia; kalmettani@kau.edu.sa

**Keywords:** S-CO_2_ Brayton cycle, exergy efficiency, irreversibility, thermal efficiency, improved heat recovery

## Abstract

This article deals with the thermodynamic assessment of supercritical carbon dioxide (S-CO_2_) Brayton power cycles. The main advantage of S-CO_2_ cycles is the capability of achieving higher efficiencies at significantly lower temperatures in comparison to conventional steam Rankine cycles. In the past decade, variety of configurations and layouts of S-CO_2_ cycles have been investigated targeting efficiency improvement. In this paper, four different layouts have been studied (with and without reheat): Simple Brayton cycle, Recompression Brayton cycle, Recompression Brayton cycle with partial cooling and the proposed layout called Recompression Brayton cycle with partial cooling and improved heat recovery (RBC-PC-IHR). Energetic and exergetic performances of all configurations were analyzed. Simple configuration is the least efficient due to poor heat recovery mechanism. RBC-PC-IHR layout achieved the best thermal performance in both reheat and no reheat configurations ($\eta_{th}\;$ = 59.7% with reheat and $\eta_{th}\;$= 58.2 without reheat at 850 °C), which was due to better heat recovery in comparison to other layouts. The detailed component-wise exergy analysis shows that the turbines and compressors have minimal contribution towards exergy destruction in comparison to what is lost by heat exchangers and heat source.

## 1. Introduction

Efficient conversion of heat to electrical power is an issue of global interest. This has led researchers to continuously strive for a better power generation cycle with improved thermodynamic performance. Recent decades have seen a rigorous development and modification in supercritical carbon dioxide (S-CO_2_) Brayton cycles due to its capability of achieving high thermal efficiencies at low to medium temperature source [1,2]. The S-CO_2_ cycle benefits from the drastic increase in the density of carbon dioxide near critical point which significantly reduces the compression work. Recompression cycles are known to take maximum advantage of this property variation of CO_2_ near the critical point, and thus they are better and more efficient than other configurations [3,4,5,6]. Furthermore, S-CO_2_ Brayton cycles are a much more viable alternative to other thermodynamics cycles due to the following advantages: (a) Improved safety as CO_2_ is a non-toxic, stable, non-combustible [7], (b) structural simplicity and compactness [8,9], (c) low capital and maintenance cost [10,11].

Supercritical carbon dioxide Brayton cycle caught attention in 2004 when Dostál [3] refined the configurations proposed by Feher [1] and Angelino [2] in the 1960s, for the application of next generation nuclear reactors. Later studies by Kulhánek and Dostál [12] and Moisseytsev and Sienicki [13] concluded that the recompression Brayton cycle with partial cooling has the highest thermal efficiency. Sarkar [14] performed energy and exergy analysis of S-CO_2_ recompression Brayton cycle optimized for nuclear reactor. He found that the effect of operating conditions is more important on recuperator irreversibility than that of turbomachines. Sarkar and Bhattacharyya [15] did sensitivity analysis to reach optimized thermal performance of S-CO_2_ recompression Brayton cycle with reheating. They observed maximum 3.5% improvement in thermal efficiency with reheating at optimum operating conditions.

Al-Sulaiman and Atif [16] investigated the thermodynamic performance of various S-CO_2_ Brayton cycles integrated with solar power tower. Their findings demonstrated that the recompression cycle has the highest thermal efficiency and the highest net power output, whereas, regenerative cycle stands second. Later, they performed detailed energy and exergy analysis of recompression cycles driven by solar thermal systems. They concluded that the highest average exergy loss occurs in heliostat field [17]. Kim et al. [18] discussed thermodynamic performance of nine S-CO_2_ bottoming power cycles with topping gas turbine cycle powered by landfill gas. They found recompression cycle is not best suited for waste heat recovery purposes due to limited fraction of heat recovery from exhaust gas. On the other hand, partial heating cycle, being simple with single compressor and turbine, has relatively higher power density. Gavic [19] investigated various options for the heat rejection from the S-CO_2_ cycle including wet cooling, dry cooling and hybrid. He found that the hybrid cooling is flexible with reduced water usage and capital cost. Sing et al. [20] developed a model for dynamic simulation of S-CO_2_ Brayton cycle heated directly via parabolic trough collectors. He found that the active control of solar collectors is necessary to maintain the supercritical operation of the cycle and to improve power output in winter. Chacartegui et al. [21] analyzed thermodynamic performance of simple and recompression Brayton cycle with and without bottoming cycle. They found that CO_2_ cycle combined with bottoming organic Rankine cycle for solar powered plants achieves significantly higher power output and efficiency in comparison to superheated steam cycles for same incident radiation.

Use of liquefied natural gas (LNG) as heat sink to improve the thermal efficiency of power cycles has also been reported in the literature [22,23]. Siddiqui et al. [24] performed energy and exergy analysis of S-CO_2_ recompression Brayton cycle in cascade arrangement with bottoming Rankine cycle exploiting cold energy of LNG to sink the heat from the bottom cycle. They considered four different working fluids for the bottoming cycle and proposed CO_2_ due to better thermodynamic performance and compactness of the system. Angelino and Invernizzi [25] investigated power cycles using LNG as heat sink, and they found CO_2_ thermal performance better than nitrogen and steam. Wang et al. [26,27] studied thermodynamic performance of S-CO_2_ power system integrated with solar energy and biomass concluding multi-energies input as a better option for efficient utilization of resources.

Literature showed lots of developments and proposals to improve the Brayton power generation cycle employing carbon dioxide in supercritical state as working fluid. This article is written in line with these efforts and puts forward a proposal with detailed thermodynamic analysis of suggested layout which targets the improvements in the heat recuperation process in the cycle, eventually increasing the cycle’s thermal efficiency. Simulations are performed in commercial software Aspen HYSYS V9 (Aspen Technology, Inc., Bedford, MA, USA). Simple Brayton cycle, Recompression Brayton cycle and Recompression Brayton cycle with partial cooling are modelled and results are validated with the literature [12,28,29]. A modification in the heat recovery process to recompression Brayton cycle with partial cooling gives a new configuration: RBC-PC-IHR. This is done by adding one extra heat exchanger in the cycle to recuperate the heat. The details of this modification are in Section 2.4. A comprehensive energy analysis of all four layouts is presented, followed by an exergy analysis. The effect of high temperature of the cycle, cycle pressure ratio, cycle minimum pressure and cycle configuration on energy and exergy efficiency is discussed.

## 2. Cycle Configurations

**Figure 1:**
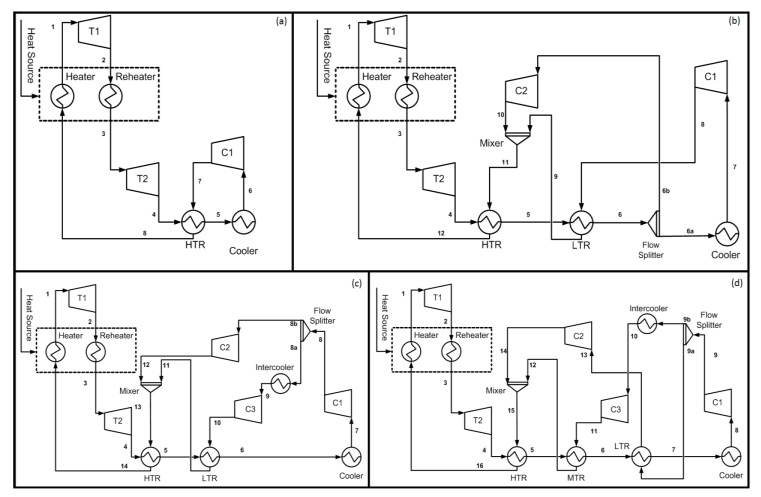
S-CO_2_ power cycle layouts: (**a**) simple Brayton cycle, (**b**) recompression Brayton cycle, (**c**) recompression Brayton cycle with partial cooling, and (**d**) recompression Brayton cycle with partial cooling and improved heat recovery. Layouts (a), (b), and (c) are adapted from [28].

In this paper, four closed-loop configurations of S-CO_2_ Brayton cycles are studied, which includes simple, recompression Brayton cycle (RBC), RBC with partial cooling (RBC-PC), and a proposed layout with improved heat recuperation. The effect of reheating is also studied for each of the above configurations, hence, altogether eight various configurations were studied. Dry cooling is used in all configurations with air being a cooling fluid. Optimal operating conditions of S-CO_2_ cycles were obtained based on first law efficiency. Figure 1 shows the layouts of each cycle and the complete description is presented in the following sections.

### 2.1. Simple Brayton Cycle (SBC)

**Figure 2:**
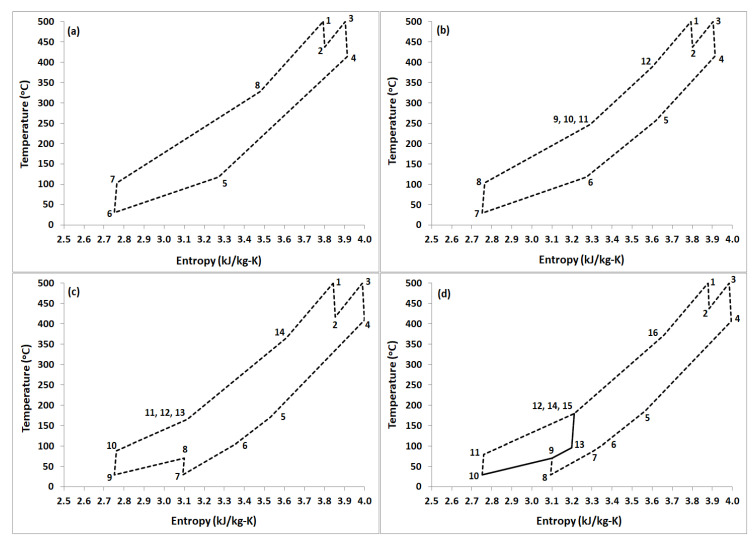
Temperature – Entropy diagram for different S-CO_2_ Brayton cycle layouts with reheat. (**a**) SBC. (**b**) RBC. (**c**) RBC-PC. (**d**) RBC-PC-IHR. High temperature of the cycle is 500 °C. All state points correspond to layouts in Figure 1.

This configuration is based on simple closed loop Brayton cycle incorporating a heat recuperator (Figure 1a). The Heat Source heats S-CO_2_ to a specified temperature in Heater and Reheater. With Reheating option on, the high pressure and high temperature S-CO_2_ (state 1) is expanded partially in turbine T1. The stream exiting T1 (state 2) is reheated and directed to Turbine T2 (state 3) where it is expanded to cycle’s low pressure. A heat recuperator recovers the heat from the stream exiting T2 (state 4) in HTR (high temperature recuperator) and preheats the stream going to the heater (state 8). The stream exiting HTR (state 5) is cooled down in a Cooler before it enters compressor C1 (state 6) where its pressure is increased to cycle’s high pressure. Part of the work output of the cycle is used to run compressor C1. In this layout, the reheat option can be turned off by setting turbine T1 expansion ratio (ER) equal to one, thus not letting the inlet stream to expand in turbine T1. Figure 2a shows the typical temperature-entropy diagram for this layout with reheat for turbine inlet temperature of 500 °C.

### 2.2. Recompression Brayton Cycle (RBC)

Swift variation in thermophysical properties of CO_2_ near critical point results in significant difference of heat capacities between hot and cold side streams in HTR. Therefore, SBC layout is restricted by the pinch point temperature in HTR [3] that limits the cycle’s thermal efficiency. The pinch point issue can be resolved by utilizing S-CO_2_ recompression Brayton cycle (RBC) configuration. This layout was originally proposed by Feher [1] and Angelio [2], then later refined by Dostál [3]. Figure 1b represents the layout used to investigate RBC. This configuration uses two recuperators (LTR, low temperature recuperator, and HTR, high temperature recuperator). The stream exiting LTR (state 6) is split into two streams. The first stream (state 6a) goes to the main compressor (C1) and the second stream (state 6b) is diverted to the recompressor (C2), which is a secondary compressor operating at the exit temperature and pressure of the LTR. Stream leaving the main compressor (state 8) is preheated in the LTR and mixed with the stream (state 10) exiting secondary compressor (C2). The mixed stream (state 11) recuperates heat through HTR before it goes to Heat Source. Part of the work output of the cycle is utilized by the compressors C1 and C2. Similar to SBC layout, the reheat option can be turned off by setting turbine T1 expansion ratio (ER) equal to one. Figure 2b displays the typical temperature-entropy diagram for this layout with reheat for turbine inlet temperature of 500 °C.

### 2.3. Recompression Brayton Cycle with Partial Cooling (RBC-PC)

Compression work in RBC can be reduced by incorporating intercooling between compression stages [30,31]. Figure 1c represents the configuration of partial cooling. The stream exiting LTR (state 6) is cooled and compressed (state 8) in the first stage of main compressor (C1). The stream is then split into two: the first stream is cooled and compressed to cycle’s high pressure (state 10) and then preheated in LTR, whereas the second stream is directly compressed to the high pressure of the cycle (state 12) and mixed with the stream (state 11) exiting LTR. The mixed stream (state 13) recovers heat in HTR and then directed to Heat Source, where it is heated to cycle’s high temperature. The rest of the flow path is identical to RBC as described earlier. Figure 2c represents the temperature-entropy diagram for this layout with reheat for turbine inlet temperature of 500 °C.

### 2.4. Recompression Brayton Cycle with Partial Cooling and Improved Heat Recovery (RBC-PC-IHR)

This layout is essentially similar to RBC-PC with slight modifications to improve the heat recuperation. This is done by introducing a third heat recuperator. In this configuration (Figure 1d), the stream leaving the LTR (state 7) is cooled (state 8) and compressed (state 9) in the first stage of main compressor (C1). The stream leaving compressor is divided into two: one stream (stream 9b) is cooled (state 10) and compressed (state 11) to cycle’s high pressure then flows to a medium temperature recuperator (MTR) to recover the heat, whereas the other stream (stream 9a) recovers heat in LTR (state 13) prior to compression to cycle’s high pressure in compressor C3. Streams leaving the compressor C2 (state 14) and MTR (state 12) are mixed together and flow to HTR to recover the heat prior to heating through Heat Source. The remaining flow path is similar to previous cycle. Figure 2d shows the temperature-entropy plot for this layout with reheat for turbine inlet temperature of 500 °C.

## 3. Energy Model

The first law efficiency, thermal efficiency, of the cycle is calculated as:

(1) $$\eta_{th}=\left({\dot{W}}_{net,\;turbine}-{\dot{W}}_{net,\;compressor}\right)/\left({\dot{Q}}_{heater}+{\dot{Q}}_{reheater}\right)$$

where ${\dot{W}}_{net,\;turbine}$ is the total work output of turbines T1 and T2, ${\dot{W}}_{net,\;compressor}$ is the total work consumed by compressors C1, C2 and C3. ${\dot{Q}}_{heater}$ and ${\dot{Q}}_{reheater}$ represent the heat input to the cycle from Heater and Reheater, respectively. 

For SBC layout, high temperature recuperator operates at a specified value of effectiveness defined as:

(2) $$\epsilon_{hot,\;stream}=\left(h_{4}-h_{5}\right)/\left(h_{4}-h_{5\left(T_{7},\;\;P_{5}\right)}\right)$$

For all remaining configurations, involving MTR and/or LTR, effectiveness is considered for the total hot stream [28,32] defined as:

(3) $$\epsilon_{hot,\;stream}=\left(h_{HTR,HI}-h_{LTR,HO}\right)/\left(h_{HTR,HI}-h_{LTR,HO@Tc}\right)$$

where $h_{HTR,HI}$ and $h_{LTR,HO}$ are the enthalpies of hot streams at the inlet of HTR and outlet of LTR, respectively. $h_{LTR,HO@Tc}$ is the enthalpy of the hot stream at the outlet of LTR, calculated based on the minimum temperature that it could achieve [33] (T_8_ for RBC, T_10_ for RBC-PC, and T_9_ for RBC-PC-IHR). Since in all configurations, except SBC, the flow stream splits into two, thus another important parameter called split ratio (SR) is introduced, which is defined as the ratio of mass flow rate of the cold stream entering LTR and the total mass flow rate of the cycle. It is equal to ${\dot{m}}_{8}/{\dot{m}}_{t}$ for RBC, ${\dot{m}}_{10}/{\dot{m}}_{t}$ for RBC-PC, and ${\dot{m}}_{9a}/{\dot{m}}_{t}\;$for RBC-PC-IHR, where ${\dot{m}}_{t}$ is the cycle’s total mass flow rate.

## 4. Simulation Environment

All S-CO_2_ Brayton cycles discussed in this paper were simulated in commercial software Aspen HYSYS V9 (Aspen Technology, Inc., Bedford, MA, USA). The Peng–Robinson model was used for calculation of state properties. The analysis was done with the following assumptions [12,14,28,29]:

1. The cycle operates under steady-state conditions.
2. Pressure and heat losses in all pipelines and equipment are considered zero.
3. The turbine and compressor adiabatic efficiencies are 93% and 89%, respectively.
4. The heat exchanger effectiveness is 95% with a minimum pinch point temperature $\left({\Delta T}_{\min}\right)\;$of 5 °C for all heat exchangers.
5. The cycle maximum pressure is 25 MPa.
6. Compressor inlet temperature and pressure are maintained at 32 °C and 7.5 MPa corresponding to state ‘6’ for SBC, state ‘7’ for RBC, state ‘9’ for RBC-PC, and state ‘10’ for RBC-PC-IHR.
7. The turbine inlet temperature varies from 500 °C to 850 °C.
8. Turbine (T1) pressure ratio (p_1_/p_2_) is set to 1.0 and 2.0, respectively, for no reheat and reheat configuration.
9. Reference temperature used in the exergy analysis is 25 °C.
10. Temperature of Heat Source is fixed at 900 °C.

## 5. Parameter Adjustments

There are a number of parameters that may significantly affect the performance of S-CO_2_ Brayton cycle, like the turbine inlet pressure and temperature, heat exchanger effectiveness, minimum allowable pinch temperature in the heat exchanger, flow split ratio for the configurations involving recompression, compressor inlet temperature and pressure [1,2,3,34,35]. This section discusses the effect these parameters have on thermodynamic efficiency of the cycle. However, some parameters were fixed to maintain the similarity with the published data in the literature, as mentioned in Section 4. 

**Figure 3:**
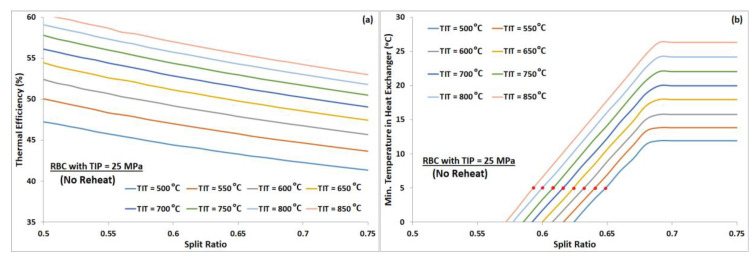
(**a**) Thermal efficiency plots of RBC (no reheat) versus split ratio, with a TIP of 25 MPa, for TIT from 500 °C to 850 °C. (**b**) Minimum pinch temperature versus split ratio; red dots indicate the minimum pinch temperature of 5 °C.

**Figure 4:**
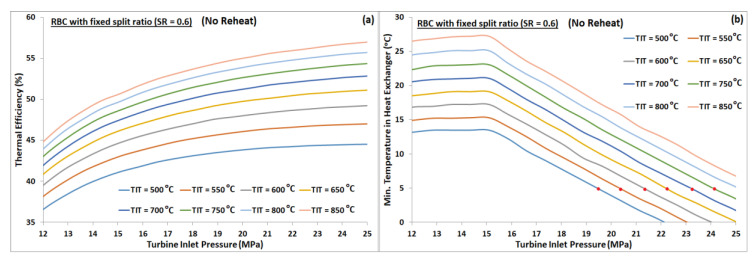
(**a**) Thermal efficiency plots of RBC-PC (no reheat) against TIP, with a fixed split ratio of 0.6, for TIT from 500 °C to 850 °C. (**b**) Minimum pinch temperature versus TIP; red dots denote the minimum pinch temperature of 5 °C.

**Figure 5:**
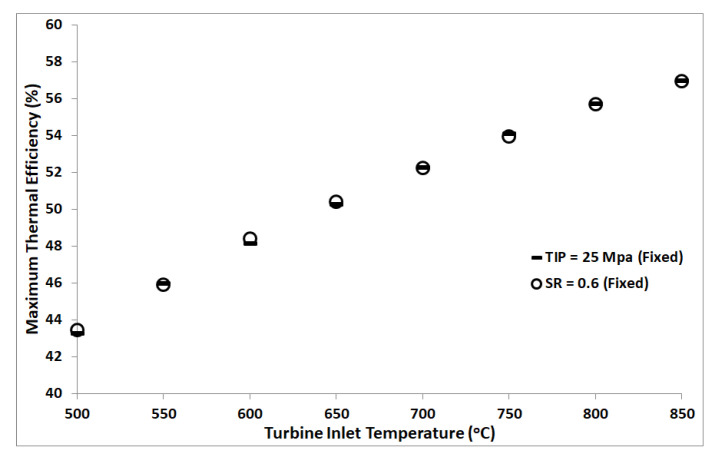
Maximum thermal efficiency plotted versus turbine inlet temperature. Data points are extracted from Figure 3a and Figure 4a corresponding to a minimum pinch temperature of 5 °C in the heat exchangers.

A study was carried out to understand the effect of split ratio for a fixed turbine inlet pressure and vice versa. RBC configuration without reheat was used for this purpose. We observed a continuous decrease in thermal efficiency with increasing split ratio for all turbine inlet temperatures, as manifested by Figure 3a. However, the minimum temperature difference between the hot and cold streams in the heat exchanger increases with increasing split ratio, see Figure 3b. Minimum pinch temperature in the current investigation was assumed 5 °C; thus, the red dots in Figure 3b indicate the near-optimal split ratio for each turbine inlet temperature. On the other hand, for a fixed split ratio, the cycle’s thermal efficiency increases monotonically with increasing turbine inlet pressure, see Figure 4a. Whereas, the minimum temperature difference between the hot and cold streams in the heat exchanger decreases, as shown by Figure 4b. Thus, the maximum operating pressure of the cycle is limited by the minimum allowable pinch temperature in the heat exchanger. Red dots in Figure 4b represent the minimum pinch temperature of 5 °C, which corresponds to near-optimal turbine inlet pressure for each turbine inlet temperature. In order to compare the effect of keeping TIP fixed while varying SR, and vice versa, on cycle’s performance, one can extract and plot the maximum thermal efficiencies from Figure 3a and Figure 4a corresponding to minimum pinch temperature of 5 °C at different turbine inlet temperatures. Figure 5 graphically represents the variation of cycle’s maximum thermal efficiency with fixed SR and with fixed PR. It is evident from this plot that one can keep either SR or PR constant while optimizing the other to maximize the cycle’s thermal efficiency.

**Figure 6:**
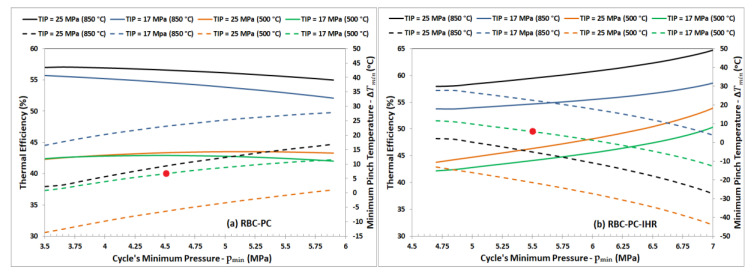
Cycle thermal efficiency (solid lines) and minimum pinch temperature in the heat exchangers (dashed lines) plotted versus cycle’s minimum pressure for (**a**) RBC-PC and (**b**) RBC-PC-IHR. Study conducted with TIP of 17 MPa and 25 MPa for TIT of 500 °C and 850 °C. The red dot indicates the near optimal value of cycle’s minimum pressure (p_7_ for RBC-PC and p_8_ for RBC-PC-IHR) that satisfies the requirement of minimum pinch temperature of 5 °C. Flow split ratio was set to 0.5 for both configurations.

Another study was done for the S-CO_2_ Brayton cycle configurations involving three compressors, i.e., RBC-PC and RBC-PC-IHR, to investigate the effect of cycle’s minimum pressure, p_min_, (p_7_ for RBC-PC and p_8_ for RBC-PC-IHR) on thermal efficiency. It was done for turbine inlet temperature of 500 °C and 850 °C with turbine inlet pressure of 17 MPa or 25 MPa. Figure 6 graphically represents the variation of the cycle’s thermal efficiency and the minimum pinch temperature as a function of cycle’s minimum pressure (p_7_ for RBC-PC and p_8_ for RBC-PC-IHR). Considering the RBC-PC configuration (Figure 6a) for TIT of 850 °C, we observe consistent decrease in thermal efficiency with increasing p_min_, however, minimum pinch temperature of 5 °C occurs at p_min_ near 4 MPa for TIP of 25 MPa. For TIT of 500 °C, the minimum pinch temperature remains negative for TIP of 25 MPa, which signifies that the temperature is crossed in the heat exchanger. On the other hand, with TIP of 17 MPa, a minimum pinch temperature condition of 5 °C satisfies near p_min_ equals to 4.3 MPa. Thus, a minimum cycle pressure for RBC-PC (p_7_) shall be 4.5 MPa for all later investigations. Similar investigation for RBC-PC-IHR configuration suggests the minimum cycle pressure (p_8_) should be 5.5 MPa. 

## 6. Simulation Validation

Simulation results obtained for SBC, RBC, and RBC-PC were validated with the results published by Padilla et al. [28], Turchi et al. [29] and Kulhánek and Dostál [12]. Table 1 lists the parameters used in the validation process.

**Figure 7:**
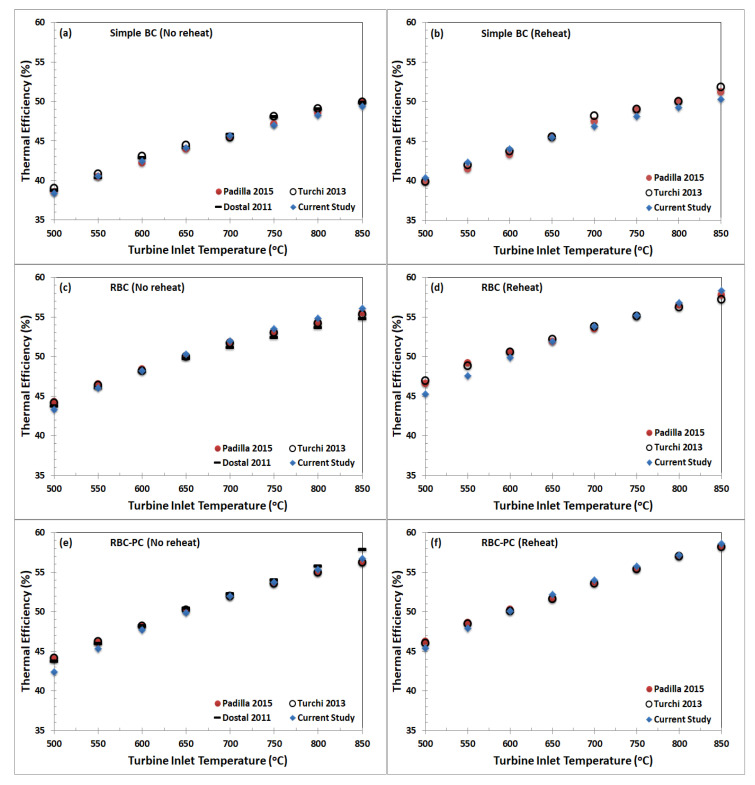
Validation of simulation results for SBC, RBC, and RBC-PC with [12], [28], [29]. Input data for the validation are listed in Table 1.

Figure 7 presents thermal efficiency for SBC, RBC, and RBC-PC plotted against turbine inlet temperature for a range of 500 °C to 850 °C. The results obtained from the developed models showed excellent agreement with the published data from [12,28,29]. This validates the simulation results and the procedures. Hence, the model developed for RBC-PC is slightly modified for better heat recovery as per configuration shown in Figure 1d. Energy and exergy analysis are carried out to assess the thermodynamic performance of proposed configuration. 

## 7. Energy Analysis of Proposed Layout (RBC-PC-IHR)

**Figure 8:**
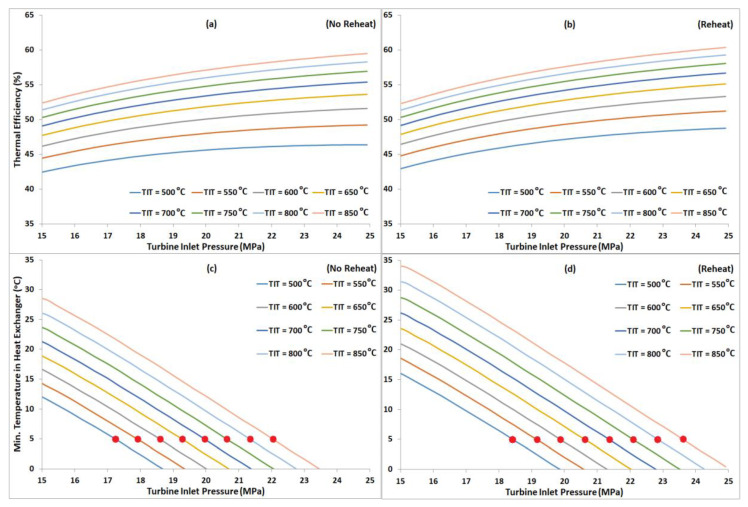
(**a**,**b**) Thermal efficiency of RBC-PC-IHR with and without reheat plotted versus TIP, with a fixed split ratio of 0.5, for TIT from 500 °C to 850 °C. (**c**,**d**) Minimum pinch temperature versus TIP; red dots denote the minimum pinch temperature of 5 °C.

**Figure 9:**
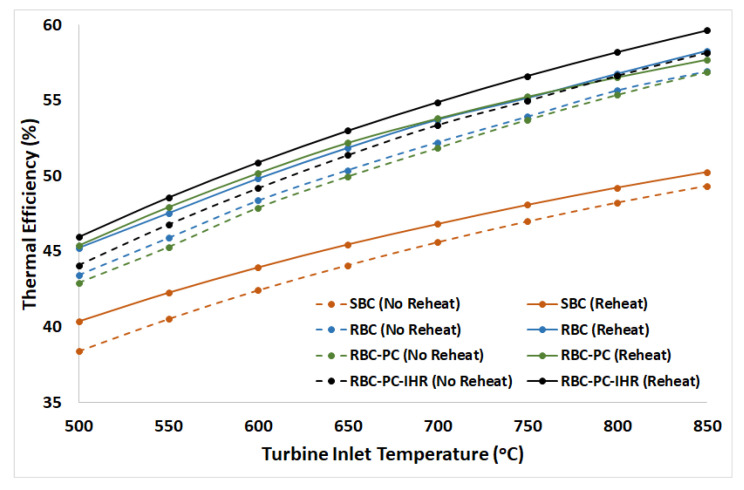
Thermal efficiency plots of different S-CO_2_ Brayton cycles for reheat and no reheat configurations. Turbine operating pressures (TIP) for each case are the same as specified in Table 2.

Energy analysis was carried out to compare the thermodynamic performance of RBC-PC-IHR (reheat and no reheat layouts) with other layouts. Figure 8a,b display the plots of thermal efficiency of RBC-PC-IHR versus TIP between 15 MPa and 25 MPa for a range of turbine operating temperatures (from 500 °C to 850 °C). Minimum pinch temperature in the heat exchangers against TIP are also shown in Figure 8c,d for each TIT. Like other configurations, a monotonic increase in thermal efficiency is observed with increasing turbine inlet pressure for all turbine inlet temperatures. However, the maximum cycle’s efficiency is limited by the allowable minimum pinch temperature in the heat exchanger, as it keeps decreasing with increasing TIP for both configurations, i.e., reheat and no reheat. In Figure 9, thermal efficiencies of all configurations are plotted versus TIT to compare their performances. SBC layout is the least efficient and RBC-PC-IHR layout is the most efficient in both configurations (with and without reheat). RBC performs slightly better than RBC-PC in no reheat configuration, however, RBC-PC recuperate heat marginally better than RBC with reheat. It is also observed that the RBC-PC-IHR layout achieves efficiencies in no reheat configuration, particularly at TIT above 700 °C, that are achieved by RBC-PC and RBC layouts with reheat only. Table 2 shows the improvement of thermal efficiency in percentage points for different configurations with respect to SBC layout, showing RBC-PC-IHR being the most efficient with an average improvement of 7.4% and 7.7% in no reheat and reheat configurations, respectively. RBC layout is marginally better than RBC-PC layout in no reheat configuration with an average improvement of 6.1%, whereas, RBC-PC perform slightly better than RBC in reheat with an average improvement of 6.6%.

## 8. Exergy Model

The exergy at each state is calculated as

(4) $${\dot{\psi }}_{j}={\dot{m}}_{j}\left(h_{j}-T_{ref}s_{j}\right)$$

where ${\dot{m}}_{j}$, $h_{j}$ and $s_{j}$ represent the mass flow rate, enthalpy and entropy of any state ‘*j*’, respectively, and $T_{ref}$ is the reference temperature. Assuming constant temperature of Heat Source ($T_{source}$), exergy input to the cycle is calculated as

(5) $${\dot{\psi }}_{input}=\left({\dot{Q}}_{Heater}+{\dot{Q}}_{Reheater}\right)\left(1-\frac{T_{ref}}{T_{source}}\right)$$

Fractional exergy loss (irreversibility) of each component of the cycle is calculated according to the following equations. The kinetic and potential energy change is neglected.

(6) $$I_{source}=\frac{\left({\dot{\psi }}_{input}-\left(\left({\dot{\psi }}_{Heater,out}-{\dot{\psi }}_{Heater,in}\right)+\left({\dot{\psi }}_{Reheater,out}-{\dot{\psi }}_{Reheater,in}\right)\right)\right)}{{\dot{\psi }}_{input}}$$

(7) $$I_{T1}=\frac{\left(\left({\dot{\psi }}_{TI,in}-{\dot{\psi }}_{T1,out}\right)-{\dot{W}}_{T1}\right)}{{\dot{\psi }}_{input}}$$

(8) $$I_{T2}=\frac{\left(\left({\dot{\psi }}_{T2,in}-{\dot{\psi }}_{T2,out}\right)-{\dot{W}}_{T2}\right)}{{\dot{\psi }}_{input}}$$

(9) $$I_{C1}=\frac{\left({\dot{W}}_{C1}-\left({\dot{\psi }}_{C1,out}-{\dot{\psi }}_{C1,in}\right)\right)}{{\dot{\psi }}_{input}}$$

(10) $$I_{C2}=\frac{\left({\dot{W}}_{C2}-\left({\dot{\psi }}_{C2,out}-{\dot{\psi }}_{C2,in}\right)\right)}{{\dot{\psi }}_{input}}$$

(11) $$I_{C3}=\frac{\left({\dot{W}}_{C3}-\left({\dot{\psi }}_{C3,out}-{\dot{\psi }}_{C3,in}\right)\right)}{{\dot{\psi }}_{input}}$$

(12) $$I_{HTR}=\frac{\left(\left({\dot{\psi }}_{HTR,HI}-{\dot{\psi }}_{HTR,HO}\right)-\left({\dot{\psi }}_{HTR,CO}-{\dot{\psi }}_{HTR,CI}\right)\right)}{{\dot{\psi }}_{input}}$$

(13) $$I_{MTR}=\frac{\left(\left({\dot{\psi }}_{MTR,HI}-{\dot{\psi }}_{MTR,HO}\right)-\left({\dot{\psi }}_{MTR,CO}-{\dot{\psi }}_{MTR,CI}\right)\right)}{{\dot{\psi }}_{input}}$$

(14) $$I_{LTR}=\frac{\left(\left({\dot{\psi }}_{LTR,HI}-{\dot{\psi }}_{LTR,HO}\right)-\left({\dot{\psi }}_{LTR,CO}-{\dot{\psi }}_{LTR,CI}\right)\right)}{{\dot{\psi }}_{input}}$$

As mentioned in the introduction, dry cooling is suggested for coolers with air being cooling fluid, therefore, a portion of exergy input to coolers is transferred to cooling air as a result of heat transfer. The exergy gain by the cooling air in each cooler can be approximated as [28]:

(15) $${\dot{\psi }}_{gain,air}={\dot{m}}_{air}{\left[\left(h_{out}-h_{in}\right)-T_{ref}\left(s_{out}-s_{in}\right)\right]}_{air}$$

The exergy loss in each cooler by CO_2_ can be calculated as [28]:

(16) $${\dot{\psi }}_{loss,CO_{2}}={\dot{m}}_{CO_{2}}{\left[\left(h_{in}-h_{out}\right)-T_{ref}\left(s_{in}-s_{out}\right)\right]}_{CO_{2}}-{\dot{\psi }}_{gain,air}$$

Therefore, total irreversibilities in the coolers combine both exergy losses by the CO_2_ and exergy losses to the environment due to heat transfer to the air:

(17) $$I_{Cooler}=\frac{{\left({\dot{\psi }}_{gain,air}\right)}_{Cooler}+{\left({\dot{\psi }}_{gain,air}\right)}_{Intercooler}+{\left({\dot{\psi }}_{loss,CO_{2}}\right)}_{Cooler}+{\left({\dot{\psi }}_{loss,CO_{2}}\right)}_{Intercooler}}{{\dot{\psi }}_{input}}$$

Net irreversibility of the cycle is obtained by adding fractional exergy losses of individual components.

The overall exergy efficiency is calculated as:

(18) $$\eta_{exergy}=\left({\dot{W}}_{net,\;turbine}-{\dot{W}}_{net,\;compressor}\right)/{\dot{\psi }}_{input}$$

Equation (18) may also be expressed in terms of total exergy loss (total irreversibility) [14]:

(19) $$\eta_{exergy}=1-I_{total}$$

It can be noted that the exergy efficiency can also be expressed as a ratio of first law (thermal) efficiency by the Carnot efficiency [36], considering sink, Cooler and Intercooler, being at constant reference temperature. 

## 9. Exergy Analysis

Exergy analysis of each S-CO_2_ Brayton cycle configuration is carried out by calculating the fractional exergy losses in component-wise following a set of equations from Equations (6)–(17). For each configuration, exergetic performance is assessed at the optimum turbine inlet pressure corresponding to cycle’s maximum thermal efficiency for turbine inlet temperatures from 500 °C to 850 °C.

### 9.1. Overall Exergy Performance

**Figure 10:**
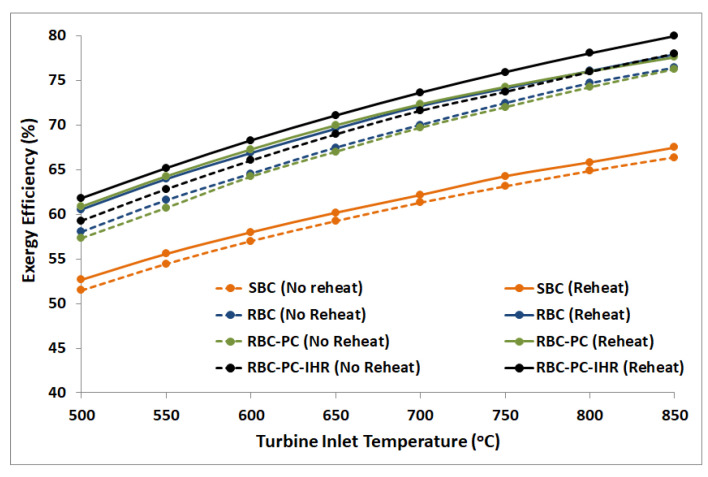
Exergy efficiency plots of different S-CO_2_ Brayton cycles for reheat and no reheat configurations. Turbine operating pressures (TIP) for each case are the same as specified in Table 2.

The cycle exergy efficiency for different S-CO_2_ Brayton cycle configurations at different turbine inlet temperatures is shown in Figure 10. All configurations display the monotonic increase in cycle’s exergy efficiency with increasing turbine inlet temperatures. It may be noted that the trend of exergy efficiency is similar to the thermal efficiency since Carnot efficiency is invariant due to constant temperature of Heat Source and reference temperature, Equation (5). For all layouts, reheat configurations show a better exergy performance in comparison to no reheat. In both reheat and no reheat configurations, SBC layout is the least efficient signifying maximum exergy loss (reheat layout:$\;52.7\%{@}_{TIT=500^{{}^{\circ}}C}-67.5\%{@}_{TIT=850^{{}^{\circ}}C}$ and no reheat layout:$\;51.5\%{@}_{TIT=500^{{}^{\circ}}C}-66.4\%{@}_{TIT=850^{{}^{\circ}}C}$). Maximum exergy performance corresponds to RBC-PC-IHR (reheat layout:$\;61.8\%{@}_{TIT=500^{{}^{\circ}}C}-80\%{@}_{TIT=850^{{}^{\circ}}C}$ and no reheat layout:$\;59.3\%{@}_{TIT=500^{{}^{\circ}}C}-78\%{@}_{TIT=850^{{}^{\circ}}C}$). Table 3 shows the improvement of overall exergy efficiency in percentage points for different configurations with respect to simple Brayton cycle layout. Results show that RBC layout (in no reheat configuration) exergy performance is slightly better than RBC-PC at lower turbine inlet temperatures; however, this gap seems to decrease with increasing turbine inlet temperatures. On the other hand, RBC-PC layout (in reheat configuration) achieves marginally greater exergy efficiency than RBC. Exergy management by RBC-PC-IHR surpasses other layouts with an average increase of 9.8% and 11.0% in no reheat and reheat configurations, respectively. 

### 9.2. Component-Wise Exergy Performance

**Figure 11:**
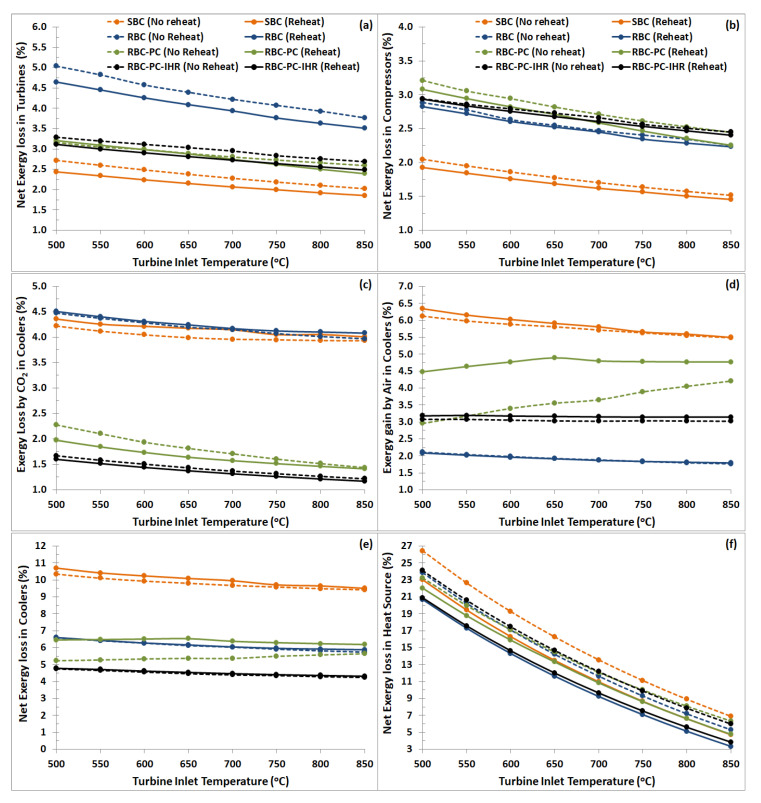
Component-wise irreversibility plotted versus turbine inlet temperatures representing (**a**) net exergy loss in turbines (T1 and T2), (**b**) net exergy loss in compressors (C1, C2 and C3), (**c**) exergy loss by CO_2_ in Cooler and Intercooler combined, (**d**) exergy gain by air in Cooler and Intercooler combined, and (**e**) net exergy loss in the Heater and Reheater together. (**f**) net exergy loss in heat source.

Fractional exergy losses in various components of the S-CO_2_ Brayton cycle for different layouts are plotted versus turbine inlet temperatures in Figure 11. Net exergy losses in turbines (T1 and T2), compressors (C1, C2 and C3), heat recovery units, i.e., recuperators (LTR, MTR and HTR), Coolers and Intercoolers, and Heater and Reheater are plotted separately to assess component-wise exergy management performance in the cycle.

#### 9.2.1. Turbines and Compressors

Figure 11a,b represent that the irreversibilities of turbo-machineries (turbines and compressors) are significantly smaller in comparison to that of heat exchangers (recuperators and coolers) and Heat Source. Figure 11a shows that the SBC configuration incurs the least net exergy losses in turbines. Modifications made to SBC layout (i.e., RBC, RBC-PC and RBC-PC-IHR) slightly increase the irreversibilities, especially for RBC; however, it is less noticeable and significant for RBC-PC and RBC-PC-IHR layouts. For all configurations, results show that the reheat layout incur prominently less exergy losses compared to no reheat which also decreases with increasing turbine inlet temperatures. The average exergy loss in turbines for reheat layout is SBC 2.13%, RBC 4.04%, RBC-PC 2.80% and RBC-PC-IHR 2.78%. In case of no reheat, it is SBC 2.35%, RBC 4.35%, RBC-PC 2.86% and RBC-PC-IHR 2.98%.

Figure 11b represents the net exergy losses occurring in all compressors for each layout. SBC layout has a single compressor (C1), thus net irreversibility due to compression is the minimum in this layout in comparison to others. RBC layout has two compressors (C1 and C2) which resulted in increased net exergy loss in compression process with respect to SBC. RBC-PC and RBC-PC-IHR layouts have three compressors (C1, C2 and C3), however, exergy loss occurring in compression process in these layouts are not significantly higher than RBC layout. Like turbines, exergy losses in compressors tend to decrease with increasing turbine inlet temperatures. The average exergy loss in compressors for reheat layout is SBC 1.67%, RBC 2.50%, RBC-PC 2.65% and RBC-PC-IHR 2.65%. In case of no reheat, it is SBC 1.76%, RBC 2.54%, RBC-PC 2.79% and RBC-PC-IHR 2.69%.

#### 9.2.2. Coolers

In all the variants of Brayton cycles, coolers (Cooler and Intercooler) are used to cool down CO_2_ stream to predefined temperature. As mentioned in an earlier section, air is used as a cooling fluid for this purpose. Thus, apart from irreversibilities due to internal heat transfer (exergy loss by CO_2_ in coolers), part of input exergy is lost to the environment as well, because air also increases its exergy as a result of heat transfer with CO_2_. Figure 11c,d show the contribution of internal and external irreversibilities to the total exergy loss occurring in the coolers. The external irreversibilities (Figure 11d) are noticeably greater than internal irreversibilities (Figure 11c), except for RBC-PC. Thus, available useful exergy lost by CO_2_ was received by the air in the air coolers (rejected to environment). The introduction of third heat exchanger (MTR) in RBC-PC-IHR takes care of heat recovery which targets to reduce the exergy gain by the air in the cycle and this is evident from Figure 11d. Net exergy losses in the coolers, internal plus external, are plotted in Figure 11e. The average exergy loss in coolers for reheat layout is SBC 10.03%, RBC 6.16%, RBC-PC 6.38% and RBC-PC-IHR 4.52%. In case of no reheat, it is SBC 9.79%, RBC 6.11%, RBC-PC 5.41% and RBC-PC-IHR 4.46%. Results show that the RBC-PC-IHR layouts experience significantly less irreversibilities in comparison to other layouts. This is a result of better heat recuperation due to additional heat exchanger in the cycle which ultimately reduces the heat exchange temperature difference in the coolers.

#### 9.2.3. Heat Source

Heater and Reheater, being the source of exergy input to the cycle, are assumed to operate at predefined fixed temperature. For each case, irreversibilities associated with Heat Source, calculated using Equation (6), are plotted in Figure 11f. It is to be noted that for the reheat case, total exergy losses occurring in Heater and Reheater combined are plotted. Results show that the exergy losses in Heat Source are most significant and dominating, especially at low turbine inlet temperatures which is due to higher heat exchange temperature difference. However, it drops significantly as the turbine inlet temperature is increased. For all configurations, addition of reheat to the cycle decreases the exergy losses; the average decrease in percentage points is SBC −2.74%, RBC −2.51%, RBC-PC −1.31% and RBC-PC-IHR −2.62%.

#### 9.2.4. Recuperators

**Figure 12:**
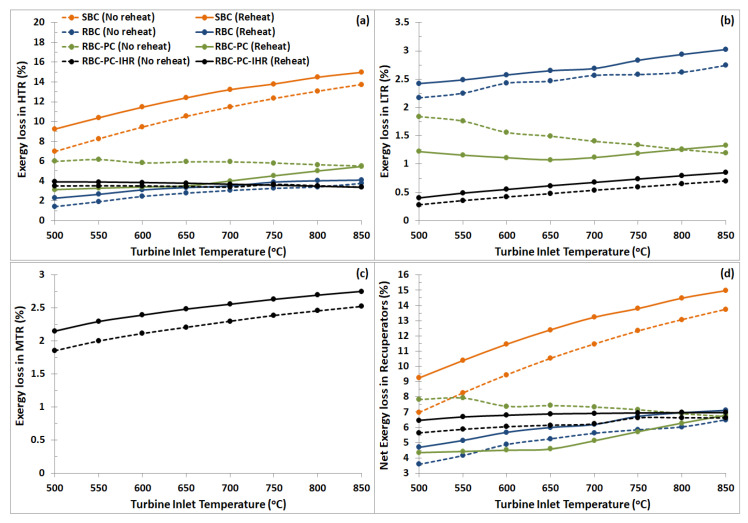
Exergy loss versus turbine inlet temperatures in SBC, RBC, RBC-PC and RBC-PC-IHR for (**a**) HTR, (**b**) LTR, (**c**) MTR and (**d**) all recuperators in the layout combined, plotted for reheat and no reheat configurations.

Exergy loss occurring during heat recovery process at various recuperators are plotted as a function of turbine inlet temperatures and shown in Figure 12. In comparison to SBC, addition of heat recovery units in other layouts (RBC, RBC-PC and RBC-PC-IHR) significantly decreases the net exergy losses in recuperators. The average decrease in percentage points for reheat configuration is RBC −6.43%, RBC-PC −7.27% and RBC-PC-IHR −5.67%. For no reheat cases, it is RBC −5.50%, RBC-PC −3.40% and RBC-PC-IHR −4.50%. This shows that the absence of second recuperator in SBC layout results in considerably high exergy losses during heat recovery process which noticeably increases with increasing turbine inlet temperature. Practically, this is a result of higher pinch temperatures in SBC layout for a fixed maximum turbine inlet pressure. This issue is resolved to a great extent in other configurations (RBC, RBC-PC and RBC-PC-IHR). 

## 10. Conclusions

Energy and exergy analysis of different S-CO_2_ Brayton cycles were performed. Four different configurations were assessed: Simple Brayton cycle (SBC), Recompression Brayton cycle (RBC), Recompression Brayton cycle with partial cooling (RBC-PC) and a proposed layout called Recompression Brayton cycle with partial cooling and improved heat recovery (RBC-PC-IHR). Modifications to RBC-PC layout were suggested to improve heat recovery in the cycle and thus improving net energetic and exergetic performance of the cycle. Following are the key outcomes and proposed concluding remarks:

- Simple Brayton cycle (SBC) has the lowest thermal efficiency which offers at least, on average, 6.5% and 6.0% (in percentage points) less thermal efficiency, respectively, for reheat and no reheat layouts, in comparison to other configurations incorporating heat recovery units. However, it is still considered an attractive option due to its simplicity and compactness.
- Exergy analysis showed that the maximum overall exergy losses occurred in SBC layout (with and without reheat), where the losses in Cooler and HTR were significantly high. This was due to ineffective recovery of exergy in HTR and associated pinch point problem.
- All configurations showed monotonic increase in the thermal efficiency with the turbine inlet temperature.
- Proposed layout, RBC-PC-IHR, had the maximum thermal efficiency and noticeably higher than RBC and RBC-PC layouts for both reheat and no reheat configurations. In comparison to RBC and RBC-PC, the average improvement of thermal efficiency from RBC-PC-IHR in percentage points was RBC (reheat) 1.2%, RBC (no reheat) 1.3%, RBC-PC (reheat) 1.1%, and RBC-PC (no reheat) 1.4%.
- Like thermal efficiency, the overall exergy efficiency of the RBC-PC-IHR layout was significantly higher. The average increase of the overall exergy efficiency in percentage points was RBC (reheat) 1.6%, RBC (no reheat) 1.4%, RBC-PC (reheat) 1.4%, and RBC-PC (no reheat) 1.8%. Component-wise exergy analysis showed that this improvement was due to considerably less exergy loss in the Cooler and Intercooler and Heat Source. Thus, it may be concluded that the introduction of the third recuperator in the proposed layout decreased the overall external irreversibilities.
- Results showed that the exergy losses occurring in the heat exchangers and heat source were significantly greater compared to that of turbo-machineries.
- For low to medium heat source temperatures, S-CO_2_ Brayton cycle is an attractive alternative to conventional steam Rankine cycles due to its higher energetic and exergetic performance and compactness.

## Figures and Tables

**Table 1:** Input parameters used for the validation of simulation results.

Turbine Efficiency	93% ^a^
Compressor Efficiency	89%
Turbine Inlet Temperature (TIT)	500–850 °C
Heat Exchanger Effectiveness ($\epsilon_{hot,\;stream})$	95%
Cycle High Pressure	25 MPa
Compressor Inlet Temperature	32 °C
Minimum Pinch Temperature ($\Delta T_{min})$	5 °C
Turbine (T1) Pressure Ratio (p_1_/p_2_)	1.0 for no reheat and 2.0 for reheat
Split Ratio	Adjusted for each TIT to maintain minimum pinch temperature of 5 °C in heat exchangers

^a^ 90% is used to match the results for RBC with published data.

**Table 2:** Thermal efficiency improvement with respect to SBC in percentage points for other S-CO_2_ BC layouts. Turbine operating pressures (TIP) for each case are also specified.

TIT	500 °C	550 °C	600 °C	650 °C	700 °C	750 °C	800 °C	850 °C	Average
Thermal efficiency (%)-No reheat									
RBC	4.9	5.5	5.7	6.2	6.4	6.5	6.6	6.7	6.1
TIP (MPa)	18.9	19.6	21.1	21.9	22.7	23.4	24.9	24.9	
RBC-PC	4.5	4.8	5.5	5.9	6.2	6.7	7.1	7.5	6.0
TIP (MPa)	12.1	12.5	13.6	14.3	15.1	16.2	17.4	18.5	
RBC-PC-IHR	5.7	6.3	6.8	7.3	7.8	8.0	8.4	8.8	7.4
TIP (MPa)	17.2	17.9	18.7	19.3	20	20.8	21.3	22.2	
Thermal efficiency (%)-Reheat									
RBC	4.9	5.3	5.9	6.4	6.9	7.1	7.5	8.0	6.5
TIP (MPa)	21.9	21.9	22.7	23.4	24.9	24.9	24.9	24.9	
RBC-PC	5.0	5.7	6.2	6.7	7.0	7.2	7.3	7.4	6.6
TIP (MPa)	20.4	21.9	23.4	24.9	24.9	24.9	24.9	24.9	
RBC-PC-IHR	5.6	6.3	6.9	7.5	8.1	8.5	9.0	9.4	7.7
TIP (MPa)	18.3	19.2	19.9	20.8	21.4	22.2	22.9	23.8	

**Table 3:** Exergy efficiency improvement with respect to SBC in percentage points for other S-CO_2_ BC layouts. Turbine operating pressures (TIP) for each case are the same as specified in Table 2.

TIT	500 °C	550 °C	600 °C	650 °C	700 °C	750 °C	800 °C	850 °C	Average
Overall Exergy efficiency (%)-No reheat									
RBC	6.6	7.1	7.6	8.2	8.7	9.3	9.8	10.0	8.4
RBC-PC	5.9	6.3	7.2	7.8	8.4	8.9	9.4	9.9	8.0
RBC-PC-IHR	7.8	8.4	9.0	9.7	10.3	10.6	11.1	11.6	9.8
Overall Exergy efficiency (%) – Reheat									
RBC	7.9	8.4	8.9	9.4	10.0	9.8	10.2	10.4	9.4
RBC-PC	8.2	8.7	9.3	9.8	10.2	10.0	10.2	10.1	9.6
RBC-PC-IHR	9.1	9.6	10.3	10.9	11.5	11.7	12.2	12.5	11.0

## Nomenclature

CO_2_	carbon dioxide	${\dot{Q}}_{\mathrm{heater}}$	heat input to Heater (kW)
LNG	liquefied natural gas	${\dot{Q}}_{\mathrm{reheater}}$	heat input to Reheater (kW)
s	specific entropy (kJ/kg K)	ER	Expansion ratio
$T_{ref}$	reference temperature (°C, K)	*Subscripts*	
$T_{source}$	source temperature (°C, K)	*j*	state point
p	pressure (kPa)	*in*	inlet
LTR	low temperature recuperator	*out*	outlet
MTR	medium temperature recuperator	*T1*	turbine 1
HTR	high temperature recuperator	*T2*	turbine 2
TIP	turbine inlet pressure (kPa)	*C1*	compressor 1
SR	split ratio	*C2*	compressor 2
PR	pressure ratio	*C3*	compressor 3
T	temperature (°C, K)	*HI*	inlet of hot stream to heat exchanger
TIT	turbine inlet temperature (°C, K)	*HO*	outlet of hot stream to heat exchanger
$\dot{W}$	power (kW)	*CI*	inlet of cold stream to heat exchanger
I	Irreversibility	*CO*	outlet of cold stream to heat exchanger
$\dot{\psi }$	exergy (kJ/kg)	*input*	input
$\eta_{\mathrm{th}}$	thermal efficiency	*source*	source
$\eta_{exergy}$	exergy efficiency	$loss,{CO}_{2}$	loss by CO_2_
${\dot{W}}_{\mathrm{net},\mathrm{turbine}}$	net power output from turbines (kW)	gain,air	gain by air
${\dot{W}}_{\mathrm{net},\mathrm{compressor}}$	net input power to compressors (kW)	*t*	total
ϵ	heat exchanger effectiveness		
*h*	enthalpy (kJ/kg)		
$\dot{m}$	mass flow rate (kg/s)		
